# Supplementary material for: Comparison of clinical practice guidelines methods to reach diagnostic test recommendations regarding diagnostic laparoscopy for endometriosis: A scoping review
Source: PLoS One. 2024 Dec 11;19(12):e0310593. doi: 10.1371/journal.pone.0310593 (PMC11633989; doi:10.1371/journal.pone.0310593)
Supplement: S2 Table — (DOCX) [file pone.0310593.s002.docx]

**Supplemental table 2. Search strategy**

| **Database** | **Search terms and filters used** |
| --- | --- |
| Trip database | Endometriosis  Filter: guidelines |
| Medline/Pubmed | #1: "Guideline" [Publication Type] OR Guideline*[TIAB] OR Recommendation*[TIAB] OR "Algorithms"[Mesh] OR Algorithm*[TIAB] OR "Disease Management"[MESH] OR Management*[TIAB] OR “clinical protocol”[TIAB] OR “clinical pathway”[TIAB] OR “good clinical practice”[TIAB]  #2: "Endometriosis"[Mesh] OR Endometrio*[TIAB] OR "Adenomyosis"[Mesh] OR Adenomyosis[TIAB]  #3:"Laparoscopy"[Mesh] OR Laparoscop*[TIAB] OR laparoendoscop*[TIAB] OR Celioscop*[TIAB] OR Peritoneoscop*[TIAB]  Filter: 2017-2023 |
| WOS | #1: TS=(Guideline* OR Recommendation* OR Algorithm* OR Management* OR “clinical protocol” OR “clinical pathway” OR “good clinical practice”) OR TI=(Guideline* OR Recommendation* OR Algorithm* OR Management* OR “clinical protocol” OR “clinical pathway” OR “good clinical practice”) OR AB=(Guideline* OR Recommendation* OR Algorithm* OR Management* OR “clinical protocol” OR “clinical pathway” OR “good clinical practice”) OR AK=(Guideline* OR Recommendation* OR Algorithm* OR Management* OR “clinical protocol” OR “clinical pathway” OR “good clinical practice”) OR KP=(Guideline* OR Recommendation* OR Algorithm* OR Management* OR “clinical protocol” OR “clinical pathway” OR “good clinical practice”)  #2: TS=(Endometrio* OR adenomyosis) OR TI=(Endometrio* OR adenomyosis) OR AB=(Endometrio* OR adenomyosis) OR AK=(Endometrio* OR adenomyosis) OR KP=(Endometrio* OR adenomyosis)  #3: TS=(Laparoscop* OR Celioscop* OR Peritoneoscop* OR laparoendoscop*) OR TI=(Laparoscop* OR Celioscop* OR Peritoneoscop* OR laparoendoscop*) OR AB=(Laparoscop* OR Celioscop* OR Peritoneoscop* OR laparoendoscop*) OR AK=( Laparoscop* OR Celioscop* OR Peritoneoscop* OR laparoendoscop*) OR KP=(Laparoscop* OR Celioscop* OR Peritoneoscop* OR laparoendoscop*)  Filter: 2017-2023 |
| SCOPUS | #1:TITLE-ABS-KEY (Guideline* OR Recommendation* OR Algorithm* OR Management* OR “clinical protocol” OR “clinical pathway” OR “good clinical practice”)  #2: TITLE-ABS-KEY (Endometrio* OR adenomyosis)  #3: TITLE-ABS-KEY (Laparoscop* OR Celioscop* OR Peritoneoscop* OR laparoendoscop*)  Filter: 2017-2023 |
| EMBASE | ('laparoscopy'/exp OR 'laparoscopy':ti,ab,kw OR 'pelvic endoscopy':ti,ab,kw OR 'peritoneoscopy':ti,ab,kw OR 'laparoscopic surgery'/exp OR 'laparoscopic surgery':ti,ab,kw OR 'laparoscopic surgical procedures':ti,ab,kw OR 'surgery, laparoscopic':ti,ab,kw OR 'surgical procedures, laparoscopic':ti,ab,kw OR 'laparoendoscopic single site surgery'/exp OR 'less (laparoendoscopic single site surgery)':ti,ab,kw OR 'laparoendoscopic single site':ti,ab,kw OR 'laparoendoscopic single site surgery':ti,ab,kw OR 'laparoscopic single site':ti,ab,kw OR 'laparoscopic single site surgery':ti,ab,kw OR 'videolaparoscopy'/exp OR 'vals (video-assisted laparoscopy)':ti,ab,kw OR 'laparoscopy, video':ti,ab,kw OR 'video laparoscopy':ti,ab,kw OR 'video-assisted laparoscopic surgery (vals)':ti,ab,kw OR 'video-assisted laparoscopy':ti,ab,kw OR 'video-laparoscopic surgery':ti,ab,kw OR 'videoassisted laparoscopy':ti,ab,kw OR 'videolaparoscopic surgery':ti,ab,kw OR 'videolaparoscopic surgical procedure':ti,ab,kw OR 'videolaparoscopic surgical technique':ti,ab,kw OR 'videolaparoscopic surgical treatment':ti,ab,kw OR 'videolaparoscopy':ti,ab,kw OR 'videolaparoscopy (vals)':ti,ab,kw) AND ('endometriosis'/exp OR 'adenomyosis externa':ti,ab,kw OR 'endometriosis':ti,ab,kw OR 'endometriosis externa':ti,ab,kw) AND ('practice guideline'/exp OR 'clinical practice guidelines':ti,ab,kw OR 'guidelines':ti,ab,kw OR 'guidelines as topic':ti,ab,kw OR 'practice guideline':ti,ab,kw OR 'practice guidelines':ti,ab,kw OR 'practice guidelines as topic':ti,ab,kw OR 'clinical pathway'/exp OR 'clinical pathway':ti,ab,kw OR 'clinical pathways':ti,ab,kw OR 'critical path':ti,ab,kw OR 'critical pathways':ti,ab,kw OR 'pathway, clinical':ti,ab,kw OR 'good clinical practice'/exp OR 'good clinical practice':ti,ab,kw OR 'clinical protocol'/exp OR 'clinical protocol':ti,ab,kw OR 'clinical protocols':ti,ab,kw OR 'clinical research protocol':ti,ab,kw OR 'algorithm'/exp OR 'algorhythm':ti,ab,kw OR 'algorism':ti,ab,kw OR 'algorithm':ti,ab,kw OR 'algorithms':ti,ab,kw OR 'disease management'/exp OR 'disease management':ti,ab,kw OR 'diseases management':ti,ab,kw OR 'disorder management':ti,ab,kw OR 'disorders management':ti,ab,kw OR 'illness management':ti,ab,kw OR 'management of disease':ti,ab,kw OR 'management of disorder':ti,ab,kw OR 'medical management':ti,ab,kw)  Filter: 2017-2023 |
| Google | (Guideline* OR Recommendation* OR Algorithm* OR Management* OR “clinical protocol” OR “clinical pathway” OR “good clinical practice”) AND (Endometrio* OR adenomyosis) AND (Laparoscop* OR Celioscop* OR Peritoneoscop* OR laparoendoscop*)  Filter: 2017-2023 |
| Google Scholar | (Guideline* OR Recommendation* OR Algorithm* OR Management* OR “clinical protocol” OR “clinical pathway” OR “good clinical practice”) AND (Endometrio* OR adenomyosis) AND (Laparoscop* OR Celioscop* OR Peritoneoscop* OR laparoendoscop*)  Filter: 2017-2023 |
| **Guide compiling agencies** | |
| Canadian Medical Association Infobase: Clinical Practice Guidelines (CPG) | Endometriosis |
| *Base Internacional de Guías GRADE* | *Endometriosis* |
| Guidelines International Network (G-I-N) | Endometriosis |
| National Guideline Clearinghouse (NGC) | Endometriosis guidelines |
| EGuideline | Endometriosis |
| Best Practice Guideline | Endometriosis |
| **Guidance development agencies** | |
| Scottish Intercollegate Guidelines Network (SIGN) | Endometriosis |
| National Institute for HealthandCare Excellence-UK (NICE) | Endometriosis |
| Australian Clinical Practice Guidelines | Endometriosis |
| New Zealand Guidelines G | Endometriosis |
| CENETEC - Centro Nacional de Excelencia Tecnológica en Salud - Mexico | Endometriosis |
| GuíaSalud. Guías de PrácticaClínica en el SistemaNacionalde Salud de España | Endometriosis |
| Instituto de Evaluación de Tecnologías En Salud e Investigación – EsSalud | Endometriosis |
| American College of Physicians Clinical Practice Guideline | Endometriosis |
