# Supplementary material for: Comparison of clinical practice guidelines methods to reach diagnostic test recommendations regarding diagnostic laparoscopy for endometriosis: A scoping review
Source: PLoS One. 2024 Dec 11;19(12):e0310593. doi: 10.1371/journal.pone.0310593 (PMC11633989; doi:10.1371/journal.pone.0310593)
Supplement: S3 Table — (DOCX) [file pone.0310593.s003.docx]

**Supplemental table 3. Excluded studies**

| **AUTHOR** | **YEAR** | **TÍTULO** | **REASON FOR EXCLUSION** |
| --- | --- | --- | --- |
| NICE | 2017 | Endometriosis: diagnosis NICE and management | Wrong outcome |
| NEW ZEALAND | 2020 | Diagnosis and Management of Endometriosis in New Zealand | Wrong outcome |
| CENETEC | 2013 | Diagnóstico y tratamiento de la endometriosis | Wrong Population |
| American College of Radiology | 2019 | Female Infertility | Wrong Population |
| Ross | 2019 | United Kingdom National Guideline for the Management of Pelvic Inflammatory Disease (2019 Interim Update) | Wrong Population |
| Grupo de Trabajo de la Conferencia de Consenso sobre el Cáncer de Ovario ESMO-ESGO | 2019 | ESMO–ESGO Consensus Conference Recommendations on Ovarian Cancer: Pathology and Molecular Biology, Early and Advanced Stages, Borderline Tumours and Recurrent Disease | Wrong Population |
| Shannon | 2017 | Opportunistic Salpingectomy and Other Methods of Risk Reduction for Ovarian/Fallopian Tube/Peritoneal Cancer in the General Population | Wrong Population |
| Jonathan | 2020 | 2018 United Kingdom National Guideline for the Management of Pelvic Inflammatory Disease | Wrong Population |
| ESHRE | 2014 | ESHRE guideline: management of women with endometriosis | Wrong Population |
| Internet | 2019 | Cuando más no es mejor: 10 'no hacer' en el manejo de la endometriosis. Una declaración de posición de ETIC * | Wrong Population |
|  | 2019 | Papel de la laparoscopia en el diagnóstico del dolor pélvico crónico | Wrong outcome |
| As-Sanie | 2019 | Assessing research gaps and unmet needs in endometriosis | Wrong outcome |
| Chapron | 2019 | Rethinking mechanisms, diagnosis and management of endometriosis | Wrong outcome |
| Cheong | 2018 | How good are the current guidelines on endometriosis? | Wrong outcome |
| European Society of Human Reproduction and Embryology | 2022 | ESHRE Guideline Endometriosis | Wrong outcome |
| Clark | 2016 | Visual endometriosis diagnosis is reliable but outpatient tests needed | Wrong outcome |
| Hirsch | 2018 | Diagnosis and management of endometriosis: a systematic review of international and national guidelines | Wrong outcome |
| Kalaitzopoulos | 2021 | Treatment of endometriosis: a review with comparison of 8 guidelines | Wrong outcome |
| Kuznetsov | 2017 | Diagnosis and management of endometriosis: Summary of NICE guidance | Wrong Population |
| Leonardi | 2020 | Endometriosis and the Urinary Tract: From Diagnosis to Surgical Treatment | Wrong Population |
| Young | 2017 | Diagnostic and treatment guidelines for gastrointestinal and genitourinary endometriosis | Wrong Population |
| European Association of Urology | 2022 | Chronic Pelvic Pain | Wrong Population |
| SIGN | 2018 | Management of epithelial ovarian cancer | Wrong Population |
| NICE | 2010 | Interventional procedure overview of laparoscopic hysterectomy (including laparoscopic total hysterectomy and laparoscopically assisted vaginal hysterectomy) for endometrial cancer | Wrong Population |
| Mayo Clinic | 2010 | GUIDELINES FOR DIAGNOSTIC LAPAROSCOPY | Wrong Population |
| Ballester | 2018 | Surgical management of deep endometriosis with colorectal involvement: CNGOF-HAS Endometriosis Guidelines | Wrong outcome |
| Busacca | 2018 | Guidelines for diagnosis and treatment of endometriosis | Wrong outcome |
| Leonardi | 2020 | Endometriosis clinical guidance during the COVID-19 pandemic | Wrong outcome |
| ESHRE Endometriosis Guideline Development Group | 2013 | Management of women with endometriosis | Wrong outcome |
| Bourdel | 2018 | Diagnostic strategies for endometriosis: CNGOF-HAS Endometriosis Guidelines | Wrong outcome |
| Burghaus | 2021 | Endometriosis: gynecological diagnosis and treatment: What should pain management specialists know? | Wrong Population |
| Collinet | 2018 | Management of endometriosis: CNGOF-HAS practice guidelines (short version) | Wrong Population |
| Fauconnier | 2018 | Epidemiology and diagnosis strategy: CNGOF–HAS Endometriosis Guidelines | Wrong Publication Type |
| Lee | 2021 | Updated guideline for clinical evaluation and management of endometriosis | Wrong outcome |
| Hwang | 2018 | Clinical evaluation and management of endometriosis: guideline for Korean patients from Korean Society of Endometriosis | Wrong outcome |
| Legendre | 2018 | New medical treatments for painful endometriosis: CNGOF-HAS Endometriosis Guidelines | Wrong outcome |
| Ulrich | 2014 | Diagnostics and treatment of endometriosis according to the S2k guidelines | Wrong outcome |
| AUSTRALIAN | 2020 | Australian clinical practice guideline for the diagnosis and management of endoemtriosis | Wrong outcome |
| CHAPLIN | 2018 | Diagnosis and management of endometriosis | Wrong outcome |
| Mayo Clinic |  | Endometriosis: diagnosis and management. | Wrong Publication Type |
| GGPO | 2020 | Guideline on the Diagnosis, Treatment, and Follow-up of Patients with Endometrial Cancer | Wrong Population |
| Marion | 2018 | Prophylactic procedures associated with gynecological surgery for the management of superficial endometriosis and adhesions. Clinical practice guidelines from the French College of Gynecologists and Obstetricians (CNGOF) | Wrong Population |
| Alkatout | 2018 | Endometriosis: A concise practical guide to current diagnosis and treatment | Wrong Population |
| Muzii | 2018 | Management of endometriosis from diagnosis to treatment: roadmap for the future | Wrong Population |
