# Supplementary material for: Comparison of clinical practice guidelines methods to reach diagnostic test recommendations regarding diagnostic laparoscopy for endometriosis: A scoping review
Source: PLoS One. 2024 Dec 11;19(12):e0310593. doi: 10.1371/journal.pone.0310593 (PMC11633989; doi:10.1371/journal.pone.0310593)
Supplement: Attachment — Submitted filename: Response to Reviewers.docx [file pone.0310593.s004.docx]

**Response to reviewers**

**Response to Reviewer 1**

| **Comments** | **Response** |
| --- | --- |
| 1. Line 53: edit, and write “to keep in mind” | We appreciate your comment and accept the suggestion.  In line 53, we accepted the change. |
| 2. Inclusion and exclusion criteria should be clarified in Methods. For example, Authors may specify whether they included abstract-only publications, Systematic Reviews and Meta-Analyses | We thank you for your comment. We made the change in the manuscript.  We specify that we include only clinical practice guidelines and exclude systematic reviews, observational studies, clinical trials, conference proceedings, letters to the editor, and case reports. |
| 3. - In Discussion, Authors should parallel the use of Laparoscopy both in diagnosis and treatment with the benefits of sclerotherapy in preserving ovarian parenchyma. Please consider: “Ronsini C, Fumiento P, Iavarone I, Greco PF, Cobellis L, De Franciscis P. Liquid Biopsy in Endometriosis: A Systematic Review. Int J Mol Sci. 2023;24(7):6116. Published 2023 Mar 24. doi:10.3390/ijms24076116” | We appreciate your comment. We added the following text in discussion: “Despite the above, although laparoscopy reports good diagnostic and therapeutic utility in the clinical recommendations, some endometriotic lesions may be missed because some implants may be very small or hidden to white light laparoscopy, so near infrared radiation (NIR) intraoperative imaging technology has proven to be a useful tool in these situations [[34]](https://www.zotero.org/google-docs/?9JcyAk). In addition to this, it has been reported that a miRNA pattern could be a promising and cost-effective tool for the diagnosis of endometriotic implants in patients with or without symptoms, mainly by liquid biopsy [[35]](https://www.zotero.org/google-docs/?9JcyAk). Therefore, these considerations should be taken into account in the development of future clinical recommendations for patients with endometriosis”. |
| 4. Line 136: edit, and write “was found” | We appreciate your comment and accept the suggestion.  In line 136, we accepted the change. |
| 5. Please provide Figure 1 in better quality. It appears blurry. | Thank you for your comment. The quality of Figure 1 was improved |

**Response to Reviewer 2**

| **Comments** | **Response** |
| --- | --- |
| 1. Please correct some typos thorughout the text (example EMABSE) | We appreciate your comment. Errata in the text were corrected |
| 2. Discussing on clinical benefit of diagnostic laparoscopy one may argue that some occult disease can be omitted at white light laparoscopy or deep lesion in the parametrial area (doi 10.1111/aogs.13866) | We appreciate your comment. We added the following text in discussion: “Despite the above, although laparoscopy and histologic examination remain the gold standard for the diagnosis of endometriosis, some endometriotic lesions may be missed because some implants may be very small or hidden to white light laparoscopy, so near infrared radiation (NIR) intraoperative imaging technology has proven to be a useful tool in these situations”. |
